# Imbalance of Circulating T_h_17 and Regulatory T Cells in Alzheimer’s Disease: A Case Control Study

**DOI:** 10.3389/fimmu.2018.01213

**Published:** 2018-06-04

**Authors:** Timo Jan Oberstein, Lava Taha, Philipp Spitzer, Janina Hellstern, Martin Herrmann, Johannes Kornhuber, Juan Manuel Maler

**Affiliations:** ^1^Department of Psychiatry and Psychotherapy, Friedrich-Alexander-University of Erlangen-Nuremberg, Erlangen, Germany; ^2^Department of Internal Medicine 3 – Rheumatology and Immunology, Friedrich-Alexander-University of Erlangen-Nürnberg, Universitätsklinikum Erlangen, Erlangen, Germany

**Keywords:** T_h_17, regulatory T cell, T_h_1, Alzheimer’s disease, mild cognitive impairment, Tau, amyloid beta

## Abstract

The neuropathological hallmarks of Alzheimer’s disease (AD), i.e., neuritic plaques and neurofibrillary tangles, consist of beta amyloid peptides (Aβ) and hyperphosphorylated Tau. These are accompanied by reactive microglia and astrocytes in the vicinity of the neuritic plaques and by changes to the peripheral immune system, e.g., an increase of the pro-inflammatory cytokines IL-1β, IL-6, and TNF-α in the peripheral blood. To address a potential involvement of peripheral T helper cell (T_h_) subsets in AD, we conducted a case control study with 54 individuals with AD dementia (*n* = 14), with mild cognitive impairment (MCI) due to AD (MCI_AD_, *n* = 14), with MCI unlikely due to AD (MCI_other_, *n* = 13), and controls without cognitive impairment (controls, *n* = 13). The proportions of CD3^+^CD8^−^IL-17A^+^IFNγ^−^ Th17 cells, CD3^+^CD8^−^IL-17A^−^IFNγ^+^ Th1 cells, and CD4^+^CD127^low^CD25^+^ regulatory T cells (T_regs_) were assessed by flow cytometry. In addition, the correlations of the proportions of T_h_ subsets to cerebrospinal fluid biomarkers were studied. CD3^+^CD8^−^IL-17A^+^IFNγ^−^ Th17 cells were significantly increased in subjects with MCI_AD_ compared to age- and sex-matched subjects with MCI_other_ and controls (MCI_AD_ mean = 1.13, SD = 0.77; MCI_other_ mean = 0.58, SD = 0.28; and controls mean = 0.52, SD = 0.22; *p* = 0.008). The proportion of CD4^+^CD127^low^CD25^+^ T_regs_ was not altered between the different groups, but it significantly positively related with the levels of total Tau and pTau181 (*r*_Treg|totalTau_ = 0.43, *p* = 0.021, *n* = 28; *r*_Treg|pTau181_ = 0.46; *p* = 0.024, *n* = 28) in subjects with AD but not in nonAD controls (*r*_Treg|totalTau_ _=_ −0.51, *p* = 0.007, *n* = 26). The increase of circulating CD3^+^CD8^−^IL-17A^+^IFNγ^−^ Th17 cells in the early stages of AD and the association of CD4^+^CD127^low^CD25^+^ T_regs_ with neurodegeneration marker Tau may indicate that the adaptive immune system relates to neuropathological changes in AD.

## Introduction

Alzheimer’s disease (AD) is the most common cause of dementia in the elderly. The neuritic plaques and neurofibrillary tangles are the neuropathological hallmarks of AD. They are accompanied by activated, cytokine-overexpressing microglia and astrocytes in the vicinity of the diffuse and neuritic plaques at early stages of progression in AD brain and by an increase of pro-inflammatory cytokines in the peripheral blood, such as IL-1β, IL-6, and TNF-α (1–6).

The blood–brain barrier (BBB) is impaired in patients with AD (7). This may facilitate the cytokine crosstalk between microglia and astrocytes with peripheral blood mononuclear cells (PBMCs) and the migration of PBMCs through the BBB. There is increasing evidence that blood or bone marrow-derived monocytes may either promote or inhibit chronic neuroinflammation in AD after crossing the BBB comparable with resident microglia (8, 9). Similarly, increased percentages of lymphocytes, both CD8^+^ cytotoxic T-cells and CD4^+^ T helper (T_helper_) cells, have been observed in brain parenchyma of patients with AD (10).

Primarily based on murine models, various T_h_ cell lineages, including T_h_1, T_h_17, and regulatory T cells (T_regs_), have been described to be associated with AD (11–20). The T_h_1 lineage is promoted by IL-12, is characterized by the transcription factor T-bet and by the secretion of IFNγ (20–22). Th17 cells are elevated in various autoimmune diseases and diseases associated with chronic inflammation (23, 24). The differentiation of the T_h_17 lineage depends on IL-12p40/IL-23p19 and cytokines reportedly increased in AD, i.e., TGF-β, IL-1, and IL-6, and is inhibited among others by IFNγ and IL-4 (25, 26). Th17 cells are characterized by the secretion of IL-17 and typically express the transcription factor RORγt. IL-17A, which forms homodimers or heterodimers with IL-17F, acts synergistically with other pro-inflammatory cytokines and recruits neutrophils and monocytes to the sites of inflammation (25). High levels of TGF-β and IL-2, however, facilitate the differentiation of T_h_ cells into regulatory T-cells (T_regs_), which dampen pro-inflammatory responses. The influence of T_regs_ on neuroinflammation has been extensively studied in mouse models of experimental autoimmune encephalomyelitis (EAE), i.e., a commonly used model for multiple sclerosis (27). In this model, T_reg_ cells are able to dampen deleterious pro-inflammatory effects, e.g., demyelination, and promote myelin regeneration directly *via* increased CCN3 expression and by immunomodulation among others *via* suppressing the Th1- and Th17 cell-mediated immune response (28–30). There is a great variety of CD4^+^ T_regs_, usually identified by the expression of CD25^+^ and the intracellular transcription factor Foxp3 or the expression of CD25 combined with low levels of CD127 (31, 32).

In the revised criteria for AD by the diagnostic guidelines for AD of the National Institute on Aging-Alzheimer’s Association (NIA-AA) workgroups, the diagnosis of AD is no longer merely based on clinical examination, neuropsychiatric tests, and exclusion of other causes for dementia. In the revised criteria, the diagnostic procedures include the detection of amyloidopathy in terms of a decrease of amyloid beta (Aβ) 42 in cerebrospinal fluid (CSF) or a positive amyloid-PET and of neurodegeneration reflected by an increase of Tau and of hyperphosphorylated Tau (pTau) or glucose hypometabolism in FDG-PET (33, 34). The detection of the CSF biomarkers Tau and pTau does not only provide a higher sensitivity and specificity for the diagnosis of AD dementia but also indicate a higher rate of progression from mild cognitive impairment (MCI) to AD dementia (35, 36).

Based on the increase of pro-inflammatory cytokines and T_h_ subsets found in AD brains, this study assesses the proportions of CD8^+^CD3^−^IL-17A^+^IFNγ^−^ Th17 cells, CD8^+^CD3^−^IL-17A^−^IFNγ^+^ Th1 cells, and CD4^+^CD127^low^CD25^+^ T_regs_ in cryopreserved PBMCs of subjects with Alzheimer’s dementia (AD dementia), MCI due to AD (MCI_AD_), MCI unlikely due to AD (MCI_other_), and subjects without cognitive impairment (controls). The associations between the percentages of the T_h_ subsets and CSF biomarkers are investigated.

## Materials and Methods

### Study Population

The study protocol was approved by the clinical ethics committee of the University of Erlangen-Nuremberg. Patients and their authorized legal representatives provided written informed consent after receiving a complete description of the study. Fifty-four individuals with AD dementia (*n* = 14), with MCI_AD_ (*n* = 14), MCI_other_ (*n* = 13), and controls (*n* = 13) frequency matched for sex, age, and years of education were enrolled in this case control study.

Each participant was examined by a psychiatrist with advanced training in neuropsychiatry and dementia research according to the protocol of the German version of Consortium to Establish a Registry for AD (CERAD) clinical assessment battery. All participants with AD as well as 19 non-demented controls were assessed with the German version of the CERAD neuropsychological battery (CERAD-NB), trail making test part A and B (TMT-A and -B), and single letter Phonemic fluency (CERAD-NB^+^). Two subjects with AD dementia had severe cognitive deficits, therefore, only the MMSE was performed. TMT-A or TMT-B were dismissed, when the time limit had been exceeded. All subjects were assessed with SPECT and CSF dementia diagnostics [analysis of Aβ 42, Aβ 40, Aβ42/Aβ40 ratio, Tau, and phospho-Tau (pThr181, pTau)]. To exclude other potential causes for dementia, routine laboratory analyses (e.g., blood count, measurement of serum electrolytes, urea, creatinine, TSH, vitamin B12, folate, and CRP), routine CSF analyses, and MRI of the head were performed. Participants with focal brain lesions on MRI were excluded from this study. Classification into possible and probable AD dementia and MCI_AD_ was performed by diagnostic guidelines for AD of the NIA-AA workgroups (33, 34). When the decrease of Aβ 42 was substituted by a decrease of Aβ42/Aβ40 ratio for the discrimination between possible and probable AD, all 13 subjects with AD dementia fulfilled the criteria for probable AD (37). MCI probable due to AD (MCI_AD_) was similarly defined by the decrease of Aβ42/Aβ40 ratio and increase of Tau/pTau181, while all other subjects with MCI (MCI_other_) had negative CSF biomarkers.

The severity of concomitant depressive symptoms was monitored by the Beck’s depression inventory II or the geriatric depression scale. The donors of the samples were enrolled in the study during the initial diagnosis, which is why none of them received treatment with acetylcholinesterase inhibitors or memantine at the time of sample acquisition.

### CSF Biomarker Diagnostics

The concentrations of Aβ 40 and Aβ 42 in CSF were measured with commercially available ELISAs from IBL International (Hamburg, Germany). Total Tau and pTau181 in CSF were assessed with an ELISA from Fujire-bio Europe (Gent, Belgium).

### Isolation of PBMCs

Whole blood was collected in EDTA containing S-Monovettes^®^ (Sarstedt, Nümbrecht, Germany). PBMCs were isolated by density gradient centrifugation with Biocoll separatin solution (Merck, Darmstadt, Germany) and cryopreserved with Mr. Frosty™ Freezing Container (Thermo Fisher Scientific, Schwerte, Germany) in RPMI (Gibco, now Thermo Fisher Scientific) with 20% v/v FBS and 10% v/v DMSO (Roth, Karlsruhe, Germany) and stored in liquid nitrogen.

### Flow Cytometry

The following fluorochrome-conjugated monoclonal antibodies were purchased from commercial vendors: PE/Dy647 conjugated anti-CD3 (clone: MEM-57, Immunotools, Friesoythe, Germany), APC conjugated anti-CD4 (clone: EDU-2, Immunotools), PE conjugated anti-CD127 APC (clone: eBioRDR5, eBioscience, now Thermo Fisher Scientific, Frankfurt, Germany), IL-17A (clone: eBio64DEC17, eBioscience), FITC conjugated anti-CD25 (clone: HI25a, Immunotools), and Alexa488 conjugated anti-IFNγ (clone: B27, BD Biosciences; Heidelberg, Germany). PE conjugated IgG1κ (eBioscience) and FITC conjugated IgG1 (clone: PPV-06, Immunotools) served as isotype controls.

Cryopreserved PMBCs were thawed, and the number of viable leukocytes was determined by the CASY^®^ TT Cell Counter + Analyzer (OLS Omni Life Science, Bremen, Germany). Cells were resuspended in RPMI1640 with 10% v/v FBS superior (Merck Millipore, Darmstadt, Germany)/1% v/v penicillin (10,000 U/ml)/streptomycin (10 mg/ml) (S/P, Gibco)/20 µg/ml DNAse (Sigma-Aldrich, Taufkirchen, Germany), spun with 500 *g* for 5 min, and cultivated in RPMI1640/10% v/v FBS/1% v/v S/P overnight in an incubator at 37°C and 5% CO_2_. For surface staining, 0.2 × 10^6^ cells were washed twice in PBS/0.1% v/v FBS and stained with combinations of APC conjugated anti-CD4, PE conjugated anti-CD127, FITC conjugated anti-CD25, and/or FITC conjugated IgG1 (1:200, each). For the staining of intracellular cytokines, 0.2 × 10^6^ cells were stimulated with RPMI/10% FBS/1% S/P with 20 µg/ml DNAse, phorbol myristate acetate (PMA) 50 ng/ml (Sigma-Aldrich), and ionomycin 500 ng/ml (Sigma-Aldrich). After 1 h, 5 µg/ml Brefeldin (Sigma-Aldrich) and 5 µg/ml Monensin (BD Biosciences) were added, and the cells were incubated for an additional 5 h. Cells were washed twice in PBS/0.1% v/v FBS, stained with PE/Dy647 conjugated anti-CD3 and APC conjugated anti-CD8 for 20 min at room temperature in the dark. Permeabilization, fixation, and staining of intracellular cytokines with Alexa488 conjugated anti-IFNγ and PE conjugated anti-IL-17A or PE conjugated IgG1κ (1:200, each) were performed with Cytofix/Cytoperm^®^ Plus Fixation/Permeabilization Kit (BD Biosciences) according to the manufacturer’s instructions. T_h_ cells from PMA and ionomycin stimulated cultured PBMCs were defined by a combination of FSC and SSC characteristics and CD3/CD8 stain, as CD4 was extensively downregulated under PMA and ionomycin stimulation as previously reported (38–40).

Analyses were performed using a Partec CyFlow^®^ Space flow cytometer (Sysmex Europe, Norderstedt, Germany) and Flomax^®^ operating sofware v2.9 (Sysmex Europe) and Kaluza Flow Cytometry Analysis Software v1.5.20207.16062 (Beckman Coulter Life Sciences, Krefeld, Germany). Viable lymphocytes were identified by their characteristics in forward scatter (FSC) and side scatter (SSC) and the percentage of viable lymphocytes in each preparation was determined by the CASY cell counting technology. The viable lymphocyte gate was based on Annexin V and propidium iodide stain. Representative dot plots are given in Figure S3 in Supplementary Material. Isotype control antibodies and single-stained samples were used periodically for color compensation and to check the settings.

### Statistical Analysis

Scores in CERAD-NB^+^ were converted to *z*-scores. Shapiro–Wilk’s test was used to test for normality. Homogeneity of variance was assessed by Levene’s test. Group comparisons were performed using Pearson’s χ^2^ for categorical variables or the Mann–Whitney *U* test or Kruskal–Wallis test followed by Dunn’s multiple comparison test in case a significant effect was observed for ordinal or not normally distributed interval variables. The ANOVA and for groups with inhomogenous variances the Brown–Forsythe test were employed for normally distributed interval variables followed by Tukey B or Dunn–Bonferroni correction in case a significant effect was observed. Pearson correlation was used to examine the relationships between variables of interest. Data analysis was performed using the SPSS statistical package (version 20.0; SPSS, Chicago, IL, USA). Quartiles are indicated as follows: 1st quartile = Q1; 3rd quartile = Q3; significance levels are indicated as follows: ***p < 0.001; **p < 0.01; *p < 0.05; and ns, not significant.

## Results

### Description of the Study Population

The groups of subjects with AD dementia, MCI probably due to AD (MCI_AD_), MCI with negative CSF biomarkers (MCI_other_), and without cognitive impairment (controls) did not differ statistically in gender [χ^2^(3) = 1.261, *p* = 0.738, *n* = 54], age [*F*(3,54) = 2.338, *p* = 0.085, *n* = 54], or education (*H* = 2.718, d.f. = 3, *p* = 0.437, *n* = 47). A detailed listing of the levels of CSF biomarkers and *z*-scores of the CERAD-NB^+^ is given in Table 1.

**Table 1:** Clinical data including cerebrospinal fluid biomarkers and *z*-scores of the CERAD-NB^+^ of the investigated groups.

	Controls				Mild cognitive impairment (MCI)_other_				MCI_AD_				Alzheimer’s disease dementia				*p*-Value
	Mean ± SD			*n*	Mean ± SD			*n*	Mean ± SD			*n*	Mean ± SD			*n*	
Age (years)	61.1 ± 5.9				63.6 ± 7.7				66.7 ± 6.6				67.0±6.5				0.085^b^
Education (years)	15.13 ± 3.8			^d^	14.50 ± 3.8			^d^	12.2 ± 4.1				13.2±4.1				0.437^c^
Sex (♀/♂)				(4/9)				(3/10)				(5/9)				(6/8)	0.738^a^

	**Q1**	**Median**	**Q3**		**Q1**	**Median**	**Q3**		**Q1**	**Median**	**Q3**		**Q1**	**Median**	**Q3**		

Tau (pg/ml)	167.0	247.0	284.5		185.0	226.0	260.0		486.8	597.5	989.5		508.0	660.0	964.3		
pTau181 (pg/ml)	25.5	35.9	45.2		38.9	43.5	51.1		88.3	99.3	117.3		82.5	98.3	126.3		
Beta amyloid 40 (pg/ml)	9,591	12,911	17,678		9,265	13,156	14,334		14,029	15,374	20,052		13,537	14,664	16,846		
Beta amyloid 42 (pg/ml)	704.7	872.4	1,226.3		689.4	900.8	1,085.8		488.6	580.0	677.9		496.1	598.8	645.0		
Ratio Aβ 42/Aβ 40 ratio Aβ 42/Aβ 40	0.069	0.075	0.078		0.069	0.074	0.078		0.030	0.036	0.043		0.034	0.038	0.042		
MMSE	−1.3	−0.3	0.6		−3.1	−1.5	−0.3		−2.8	−2.3	−1.6		−5.3	−4.7	−3.6		
Semantic verbal fluency test	−0.5	0.2	0.8	^d^	−2.2	−1.7	−0.8	^d^	−1.6	−1.2	0.3		−2.4	−2.3	−1.1	^d^	
Boston naming test	0.3	0.5	0.7	^d^	−0.7	0.3	0.4	^d^	−1.8	−0.4	1.2		−2.8	−1.8	−1.2	^d^	
Word list memory	0.0	0.5	1.0	^d^	−2.3	−1.3	−0.2	^d^	−3.7	−2.6	−2.0		−5.1	−3.6	−3.3	^d^	
Word list recall	0.3	0.7	1.4	^d^	−1.7	−1.3	−0.4	^d^	−3.6	−2.2	−1.3		−4.3	−3.6	−2.8	^d^	
Constructional practice	0.4	0.5	0.7	^d^	0.2	0.4	0.7	^d^	−1.7	0.4	0.7		−3.7	−2.9	−1.9	^d^	
Recall of constructional praxis	−1.3	0.7	0.9	^d^	−2.0	−1.3	0.0	^d^	−3.5	−1.6	−1.3		−4.3	−3.4	−2.7	^d^	
Phonetic verbal fluency test	−0.1	0.7	2.0	^d^	−1.6	−1.2	−0.4	^d^	−0.9	−0.2	0.7		−3.1	−1.2	−0.4	^d^	
Trail making test A	−0.3	1.0	1.5	^d^	−1.4	−0.5	−0.1	^d^	−1.2	−0.3	0.6		−2.7	−2.1	−1.1	^d^	
Trail making test B	−0.5	0.5	2.2	^d^	−0.9	−0.7	−0.3	^d^	−2.1	−1.6	0.1	^d^				^d^	

*n, number of subjects; Q1, first quartile; Q3, third quartile*.

*p-Values from ^a^Person χ^2^, ^b^one-way ANOVA, ^c^Kruskal–Wallis test; ^d^data are missing for one or more subjects*.

### Circulating CD3^+^CD8^−^IL-17A^+^IFNγ^−^ T_h_17 Cells Were Increased in Participants With MCI_AD_ Compared to Subjects Without Cognitive Impairment and With MCI_other_

**Figure 1:**
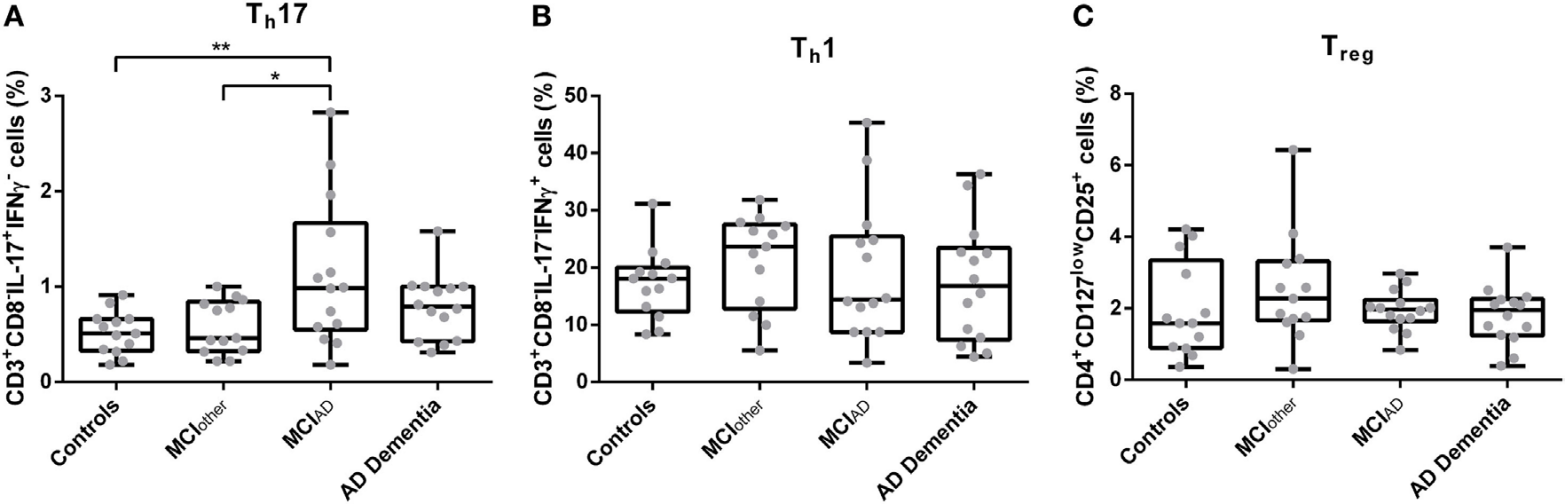
Percentages of CD3^+^CD8^−^IL-17^+^IFNγ^−^ Th17 cells **(A)**, CD3^+^CD8^−^IL-17^−^IFNγ^+^ Th1 cells **(B)**, and CD4^+^CD127^low^CD25^+^ T_regs_ **(C)** as assessed with flow cytometry from donor derived cryopreserved peripheral blood mononuclear cells were compared between controls, mild cognitive impairment (MCI)_other_, MCI_AD_, and Alzheimer’s disease (AD) dementia. Data are summarized as combined box- and scatter plots (*p*-values from *post hoc* tests are shown *p < 0.05, **p < 0.01).

The gating strategy for the analysis of CD3^+^CD8^−^IL-17A^+^IFNγ^−^ Th17 cells, CD3^+^CD8^−^IL17A^−^IFNγ^+^ T_h_1, and CD4^+^CD127^low^CD25^+^ T_regs_ from cultured cryopreserved PBMCs is depicted in Figure S1 in Supplementary Material. Doublet exclusion by FSC-H and FSC-A scatter did not relevantly alter the percentages of CD3^+^CD8^−^IL-17A^+^IFNγ^−^ T_h_17 cells, CD3^+^CD8^−^IL17A^−^IFNγ^+^ T_h_1 and CD4^+^CD127^low^CD25^+^ T_regs_ (Figure S3 in Supplementary Material). The proportion of peripheral PMA/ionomycin treated CD3^+^CD8^−^IL-17A^+^IFNγ^−^ Th17 cells was significantly altered between subjects with AD dementia, with MCI_AD_, with MCI_other_ and in controls [F*(d.f.1 = 3, d.f.2 = 24.339) = 5.314, *p* = 0.008, *n* = 54] (Figure 1). The highest abundance of CD3^+^CD8^−^IL-17A^+^IFNγ^−^ Th17 cells in relation to CD3^+^CD8^−^ cells was detected in the group with MCI_AD_ (mean = 1.13, SD = 0.77), followed by AD dementia (mean = 0.79, SD = 0.33), MCI_other_ (mean = 0.58, SD = 0.28), and controls (mean = 0.52, SD = 0.22). The subsequent Dunn–Bonferroni test showed that the proportion of CD3^+^CD8^−^IL-17A^+^IFNγ^−^ Th17 cells in MCI_AD_ was significantly elevated compared to controls (*z* = −0.61231, *p* = 0.007; *d*_Cohen_ = 1.07) and subjects with MCI_other_ (*z* = −0.55007, *p* = 0.019; *d*_Cohen_ = 0.95), indicating that circulating CD3^+^CD8^−^IL-17A^+^IFNγ^−^ Th17 cells are elevated especially in early stages of AD. Representative dotplots of CD3^+^CD8^−^ and CD4^+^ lymphocytes are given in Figure S2 in Supplementary Material.

The abundances of CD3^+^CD8^−^IFNγ^+^IL-17A^−^ Th1 cells and CD4^+^CD127^low^CD25^+^ T_regs_ from peripheral blood did not differ statistically significantly between the groups [*F*(3,50) = 0.498, *p* = 0.686, *n* = 54 and F*(3,50) = 1.106, d.f.1 = 3, d.f.2 = 34.213, *p* = 0.360, *n* = 54, respectively]. Similarly, the mean fluorescence intensities of CD3^+^CD8^−^IL-17A^+^IFNγ^−^ Th17 cells, CD3^+^CD8^−^IFNγ^+^IL-17A^−^ Th1 cells, and CD4^+^CD127^low^CD25^+^ T_regs_ did not significantly vary between AD dementia, MCI_AD_, MCI_other_, and controls [*F*(3,50) = 0.274, *p* = 0.844, *n* = 54; *H* = 1.290, d.f. = 3, *p* = 0.731, *n* = 54; and *H* = 1.669, d.f. = 3, *p* = 0.644, *n* = 54, respectively]. Analyses of control parameters showed a significant difference in the serum cholesterol levels between the groups. The other control parameters, i.e., leukocyte count, levels of CRP, of TSH, of folate, of vitamin B12, of urea, of creatinine, and of fastening glucose did not differ statistically significantly in multivariate analyses as given in Table S1 in Supplementary Material.

### The Proportion of Circulating CD4^+^CD127^low^CD25^+^ T_regs_ Cells Was Positively Related to Total Tau, pTau181, and Aβ 40 in the AD but Not in the nonAD Group

Correlations of the proportions of CD3^+^CD8^−^IL-17A^+^IFNγ^−^ Th17 cells, CD3^+^CD8^−^IFNγ^+^IL-17A^−^ Th1 cells, and CD4^+^CD127^low^CD25^+^ T_regs_ cells with total Tau, pTau181, Aβ 42, Aβ 40, and Aβ42/Aβ40 ratio were analyzed in subjects with probable AD and in subjects with negative AD biomarkers (nonAD group).

**Figure 2:**
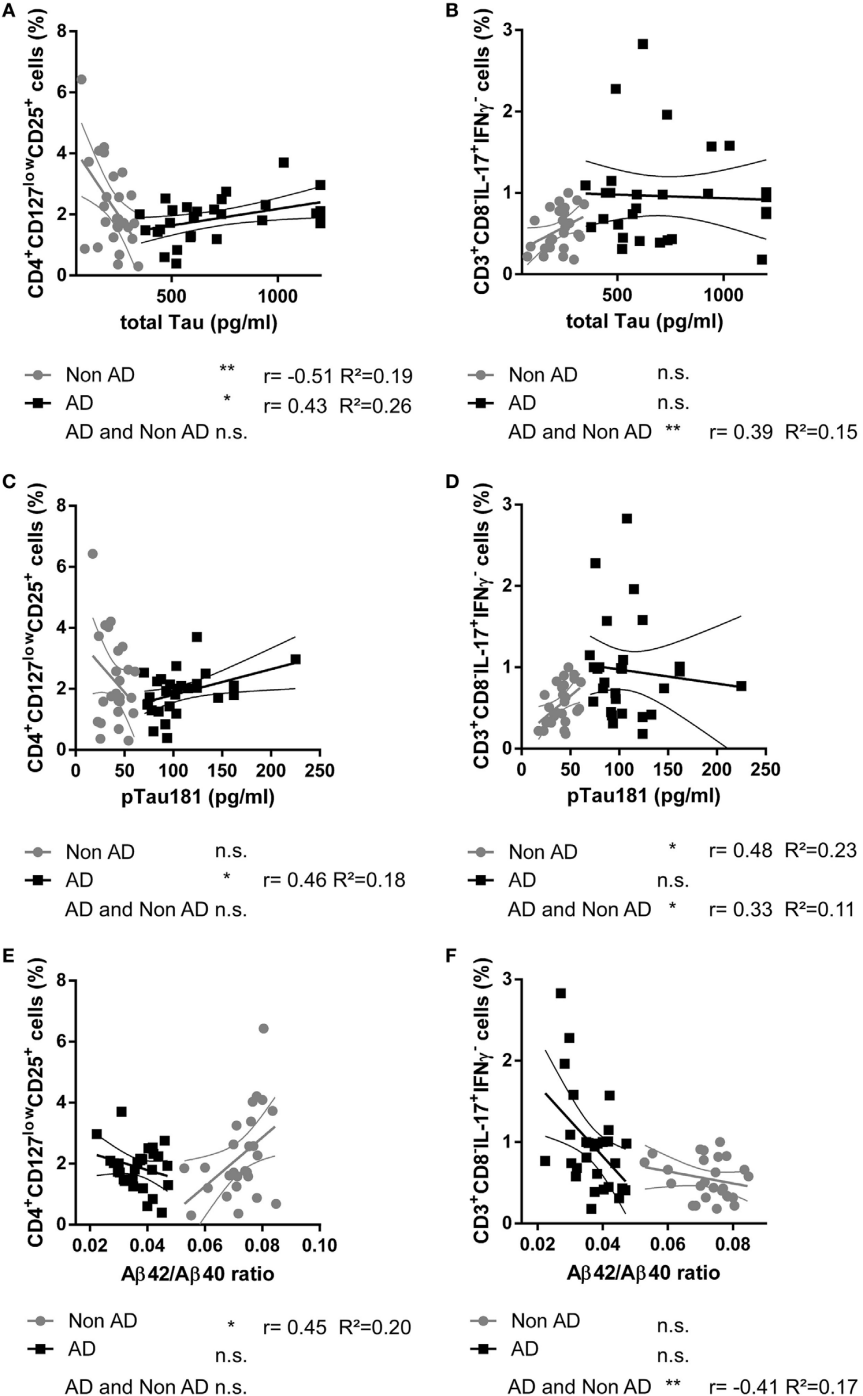
Scatter plots showing the relationship between the cerebrospinal fluid level of total Tau protein [pg/ml; **(A,B)**], pTau181 [pg/ml; **(C,D)**], Aβ42/Aβ40 ratio **(E,F)**, and the percentage of CD4^+^CD127^low^CD25^+^ T_regs_ **(A,C,E)** and CD3^+^CD8^−^IL-17^+^IFNγ^−^ Th17 cells **(B,D,F)** in donors with Alzheimer’s disease (AD, black squares) and controls (nonAD, gray dots). The associations in AD group, nonAD group and all subjects combined were investigated with Spearman’s correlation. (Scatter plots with the linear regression lines for AD and nonAD groups and their 95% confidence intervals are shown; non-significant = n.s., *p < 0.05, **p < 0.01, **p < 0.001; *r*: Spearman’s correlation coefficient; *R*^2^: coefficient of determination.)

In the AD group, the proportions of peripheral CD4^+^CD127^low^CD25^+^ T_regs_ were statistically significantly positively related to the amount of the neuronal injury marker total Tau in CSF (*r* = 0.433, *p* = 0.021, *n* = 28), to pTau (*r* = 0.462, *p* = 0.024, *n* = 28), and to Aβ 40 (*r* = 0.484, *p* = 0.009, *n* = 28) (Figure 2). In the nonAD group on the other hand, total Tau in CSF significantly negatively related to the level of CD4^+^CD127^low^CD25^+^ T_regs_ (*r* = −0.513, *p* = 0.007, *n* = 26). In addition, the percentages of CD4^+^CD127^low^CD25^+^ T_regs_ were only statistically significantly positively associated with the ratio of Aβ42/Aβ40 in the nonAD group (*r* = 0.450, *p* = 0.021, *n* = 26). Collectively, the percentage of CD4^+^CD127^low^CD25^+^ T_regs_ in the AD group positively related to the level of neurodegeneration in means of increased levels of Tau/pTau181 and amyloidopathy in means of decrease of Aβ42/Aβ40 ratio, while in the nonAD group an inverse relation was observed.

The percentages of CD4^+^CD127^low^CD25^+^ T_regs_ did not correlate with age (*r*_AD_ = 0.01, *p*_AD_ = 0.98 and *r*_nonAD_ = 0.02, *p*_nonAD_ = 0.91) or education (*r*_AD_ = 0.25, *p*_AD_ = 0.21 and *r*_nonAD_ = 0.19, *p*_nonAD_ = 0.41). There was no significant difference in the proportion of CD4^+^CD127^low^CD25^+^ T_regs_ between males and females [χ^2^(25)_AD_ = 28.000, *p*_AD_ = 0.411, *n*_AD_ = 28 and χ^2^(25)_nonAD_ = 27.000, *p*_nonAD_ = 0.356, *n*_nonAD_ = 27]. The percentage of CD3^+^CD8^−^IL-17A^+^IFNγ^−^ T_h_17 was statistically significantly positively related to pTau181 in the nonAD group (*r* = 0.480, *p* = 0.013, *n* = 26).

## Discussion

In this case control study, the proportion of peripheral CD3^+^CD8^−^IL-17A^+^IFNγ^−^ Th17 cells was significantly elevated in subjects with MCI_AD_ compared to controls and subjects with MCI_other_. Together with the observation, that the proportion of CD4^+^CD127^low^CD25^+^ T_regs_ was significantly positively related with the level of neurodegeneration markers pTau181 and total Tau in patients with AD but not in controls, the results indicate that these T_h_ cell lineages might be associated with the neurodegeneration in AD.

First indications, that the proportion of Th17 cells and RORγt^+^ cells, respectively, was elevated in Alzheimer’s dementia, were reported by Saresella et al. (16) and Agnes et al. (41). Both used the NINCDS ADRA criteria for the diagnosis of Alzheimer’s dementia without the detection of the CSF biomarkers. Saresella et al. similarly detected the highest proportions of RORγt^+^ cell in subjects with MCI, though no differentiation was made between subjects with MCI probably due to AD and MCI unlikely due to AD. An elevation of CCR6^+^ lymphocytes in 16 patients with AD has been described by Goldeck et al. (42). CCR6^+^ is a chemokine receptor found on Th17 cells, subsets of T_regs_, and dendritic cells and among others is involved in the recruitment of dendritic cells and T cells to the site of inflammation (43). In this study, we were able to differentiate the proportions of Th17 cells between MCI_AD_ and MCI_other_ for the first time. In addition, due to the simultaneous detection of the CSF biomarkers used for the diagnosis of AD dementia and MCI_AD_, we were able to relate the levels of CSF biomarkers to the percentages of CD3^+^CD8^−^IL-17A^+^IFNγ^−^ T_h_17 and CD4^+^CD127^low^CD25^+^ T_regs_. In our study, the level of Th17 cells statistically significantly related to amyloidopathy, i.e., the decrease of the ratio of Aβ42/Aβ40. Findings from previous studies indicate that Aβ can directly influence the cytokine expression of Th17 cells, as the disruption of the BBB by hippocampal application of Aβ 42 in rats was followed by an infiltration of Th17 cells into the brain parenchyma, increased expression of IL-17 and IL-22 in the hippocampus, and elevated concentrations of the two cytokines in both CSF and serum (20). The production of IL-17 from mice splenocytes was elevated after treatment with Aβ 42 and the level of IL-17A and of Th17 cells in human PBMCs was increased after the stimulation with Aβ 25–35 (13, 19).

The role of T_regs_ in the pathogenesis of AD is a matter of recent debate. In accordance with previous studies, we did not detect a significant difference between CD4^+^CD127^low^CD25^+^ T_regs_ in subjects with AD dementia or MCI_AD_ compared to age-matched controls or MCI_other_ (15, 17). However, Saresella et al. reported an increase of CD4^+^Foxp3^+^ T_regs_ in Alzheimer’s dementia especially in MCI diagnosed by NINCDS ADRA criteria, Le Page et al. reported an increase of CD4^+^Foxp3^+^CD25^high^ cells in amnestic MCI but not in mild Alzheimer’s dementia, and Larbi et al. reported an overall decreased frequency of CD4^+^CD25^high^ T cells in subjects with mild AD dementia when compared with age-matched controls (17, 44, 45).

Our findings suggest that in AD but not in controls the number of CD4^+^CD127^low^CD25^+^ T_reg_ cells was rather related to the level of Tau and pTau181, which predict the rate of cognitive decline in the different stages of AD and correlated with neurofibrillary tangle pathology in the neocortex in AD brains (35, 36, 46, 47). A possible pathomechanism might be that the number of peripheral T_regs_ was elevated in case of increased neurodegeneration in AD to prevent further demyelination and axonal loss similar to the findings in the models of EAE. Observations in a transgenic mouse model of AD (APPPS1 mice) likewise indicated that T_regs_ are able to slow the disease progression and restore cognitive function (48). In addition, an increased suppressive activity of T_reg_ cells in AD and Parkinson’s disease has been described (15). However, in 5XFAD mice carrying five mutations associated with early onset familial AD, the depletion of Foxp3^+^ T_regs_ was accompanied with increased Aβ clearance and reversal of cognitive decline and in a model for neuronal injury in BALB/c/OLA mice, the transfer of CD4^+^CD25^+^ T_regs_ worsened the outcome, indicating a deleterious effect of T_regs_ in these models (49, 50). Taken together, the function or loss of function of T_regs_ in AD has still to be determined and further studies are required to identify causal relationship and to provide evidence that the relations to the CSF biomarkers in this study are not due to a common response variable.

Complementing but further complicating the immunological changes in AD, the profile of interleukins supports our study’s findings, such as IL-6 and TNF-α, which are commonly elevated in serum in AD, are induced by IL-17 from the Th17 cells (5, 25). Furthermore, the inhibition of the IL-12/IL-23 pathway, which supports the differentiation of Th17 cells, attenuated AD pathology and cognitive deficits in an AD mouse model (51). IL-17 has been reported to be increased in AD by several reports; however, Doecke et al. detected on the contrary a significant decrease of IL-17 in plasma from AD patients in a larger study with 151 subjects (52–55). For the level of TGF-β, studies regularly reported increased as well decreased serum levels in AD (56–58). In low doses, TGF-β promotes the differentiation of Th17 cells, whereas high levels promote T_regs_, which dampen the pro-inflammatory response of Th17 cells. Interestingly, low levels of TGF-β were associated with a faster progression from MCI to AD dementia, as it was observed for high levels of total Tau and pTau181 (35, 36, 59).

Nevertheless, this study has limitations, which include the lack of a healthy control sample because of the difficulties associated with collecting CSF from healthy subjects. Instead, patients without cognitive impairment were enrolled as control. Furthermore, the small sample size and a potentially skewed population due to the fact that subjects were enrolled from our memory unit or patients hospitalized in our psychiatric ward may have some bearing on our results. A larger population study will be informative to further elaborate our initial findings.

## Conclusion

Our clinical data showed an increase of circulating CD3^+^CD8^−^IL-17A^+^IFNγ^−^ Th17 cells in subjects with MCI_AD_ and an association of CD4^+^CD25^+^CD127^low^ T_regs_ with the neurodegeneration markers phospho and total Tau, which complements the observations from fundamental research, that the adaptive immune system seems to be involved in the pathogenesis of AD and its neuropathological changes especially in the early stages of the disease. Further studies might provide more insight into disease progression and the interplay between the neuropathological hallmarks of AD and the peripheral immune system.

## Datasets are Available on Request

The raw data supporting the conclusions of this manuscript will be made available by the authors, without undue reservation, to any qualified researcher.

## Ethics Statement

The study protocol was approved by the clinical ethics committee of the University of Erlangen-Nuremberg (project no. 3987). Patients and their authorized legal representatives provided written informed consent after receiving a complete description of the study.

## Author Contributions

TO designed the study, performed experiments, analyzed the data, and together with JM drafted the manuscript. LT and JH performed experiments and contributed to revision of the manuscript. JK provided reagents and contributed to the interpretation of findings and revision of the manuscript. PS and MH contributed to the interpretation of findings and revision of the manuscript. All the authors read and approved the final manuscript.

## Conflict of Interest Statement

The authors declare that the research was conducted in the absence of any commercial or financial relationships that could be construed as a potential conflict of interest.
