# Supplementary figures and images for: Imbalance of Circulating Th17 and Regulatory T Cells in Alzheimer’s Disease: A Case Control Study
Source: Front Immunol. 2018 Jun 4;9:1213. doi: 10.3389/fimmu.2018.01213 (PMC5994416; doi:10.3389/fimmu.2018.01213)

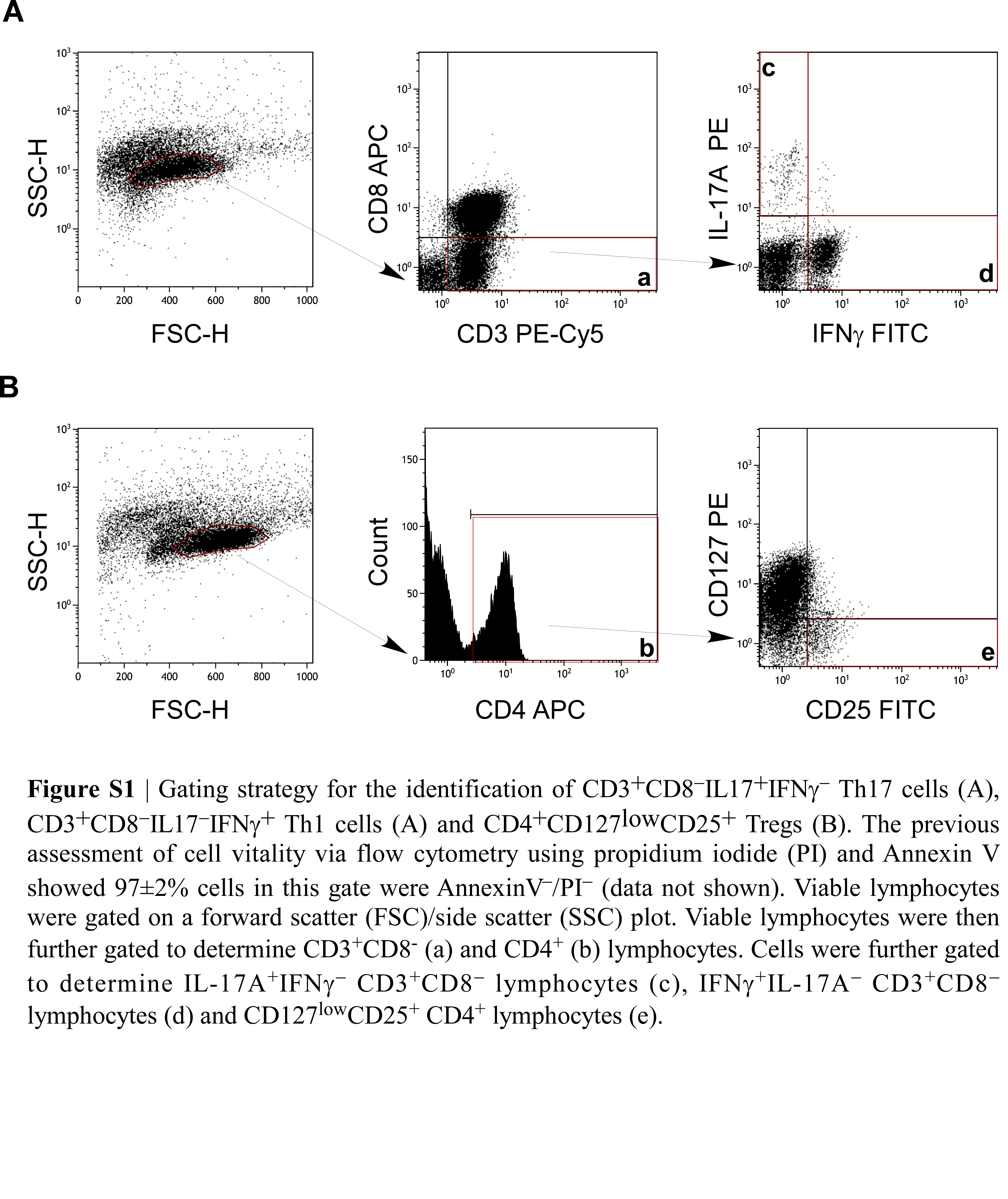

Supplement: Supplementary file 1 [file image_1.tif]

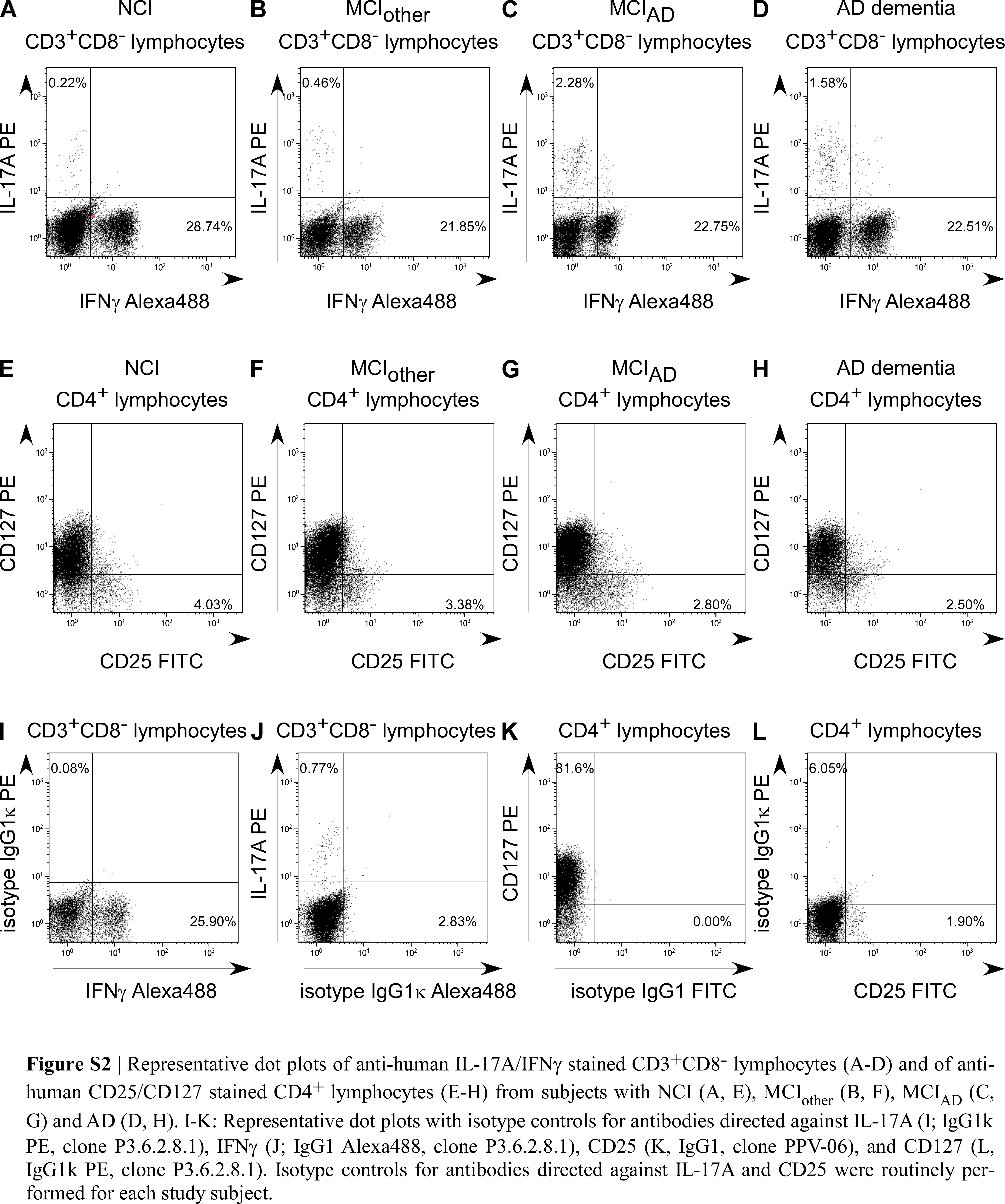

Supplement: Supplementary file 2 [file image_2.tif]

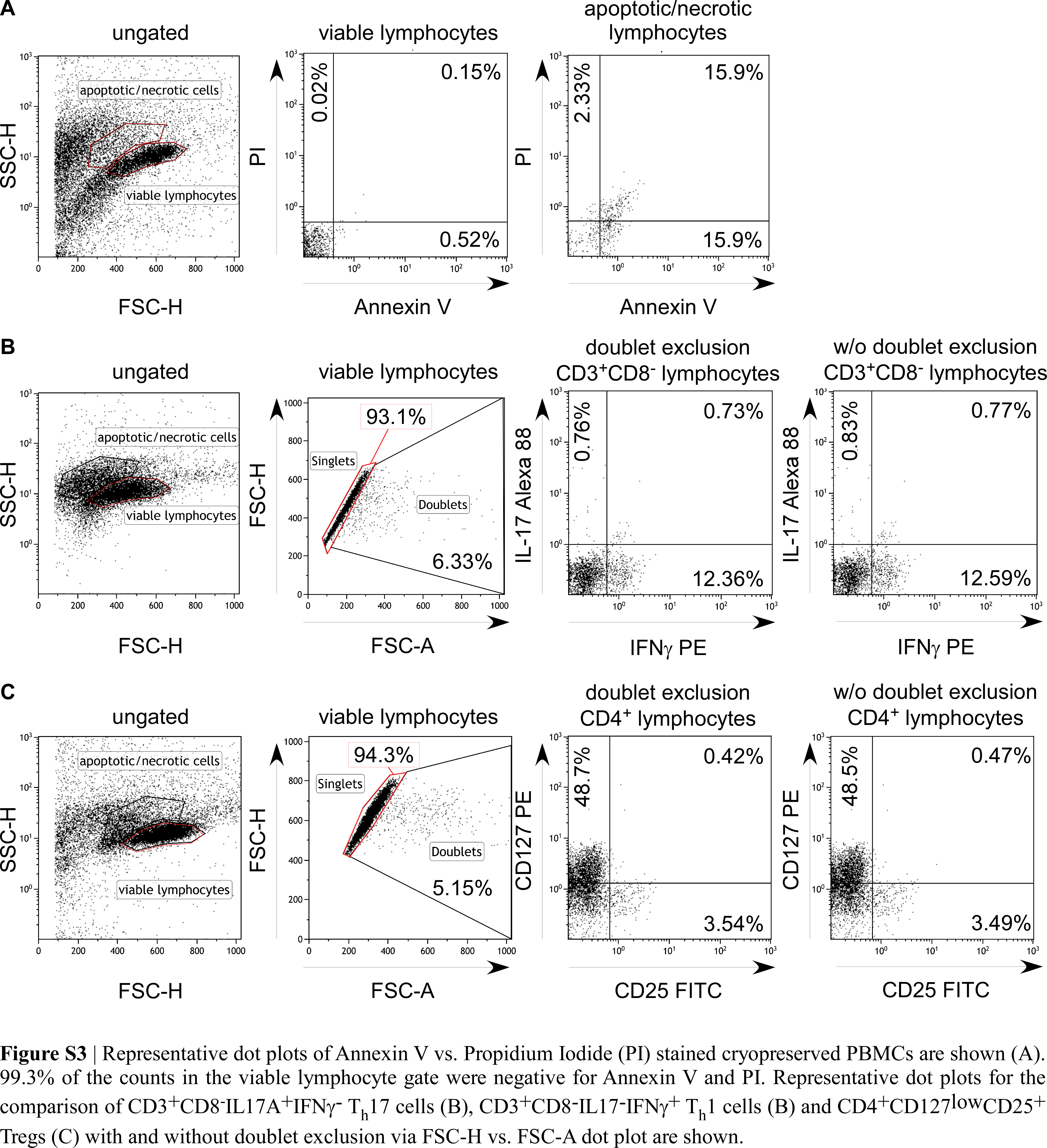

Supplement: Supplementary file 3 [file image_3.tif]
